# Seed Priming Based on Iodine and Selenium Influences the Nutraceutical Compounds in Tomato (*Solanum lycopersicum* L.) Crop

**DOI:** 10.3390/antiox12061265

**Published:** 2023-06-13

**Authors:** Fernando Mejía-Ramírez, Adalberto Benavides-Mendoza, Susana González-Morales, Antonio Juárez-Maldonado, Francisco Marcelo Lara-Viveros, América Berenice Morales-Díaz, Álvaro Morelos-Moreno

**Affiliations:** 1 Department of Horticulture, Universidad Autónoma Agraria Antonio Narro, Saltillo 25315, Mexico; 2 National Council of Humanities, Science and Technology (CONAHCYT), Universidad Autónoma Agraria Antonio Narro, Saltillo 25315, Mexico; 3 Department of Botany, Universidad Autónoma Agraria Antonio Narro, Saltillo 25315, Mexico; 4 Department of Biosciences and Agrotechnology, Centro de Investigación en Química Aplicada (CIQA), Saltillo 25294, Mexico; 5 Robotics and Advanced Manufacturing, Centro de Investigación y de Estudios Avanzados (CINVESTAV), Ramos Arizpe 25900, Mexico

**Keywords:** KIO_3_, Na_2_SeO_3_, antioxidant, seed imbibition, tomato

## Abstract

The use of trace elements in agriculture as a complement to crop fertilization programs is a practice that is gaining importance and relevance worldwide. Iodine and selenium perform essential functions in human health, related to the proper functioning of the thyroid gland, acting as antioxidants and antiproliferatives, and their limited intake through food consumption can cause malnutrition, reflected in the abnormal development and growth of humans. This research aimed to evaluate the nutraceutical quality of tomato (*Solanum lycopersicum* L.) in response to seed priming based on KIO_3_ (0, 100, 150, 200, 250 mg L^−1^) and Na_2_SeO_3_ (0, 0.5, 1, 2, 3 mg L^−1^), performed by interaction from a 5^2^-factorial design and by independent factors in a 24-h imbibition time. The tomato crop was established under greenhouse conditions in 10-L polyethylene containers containing peat moss and perlite 1:1 (*v*/*v*). Regarding non-enzymatic antioxidant compounds, lycopene, β-carotene and flavonoid contents in tomato fruits significantly increased with KIO_3_ and Na_2_SeO_3_ treatments; however, vitamin C content was negatively affected. KIO_3_ increased the phenol and chlorophyll-*a* contents of leaves. In relation to enzymatic activity, KIO_3_ positively influenced GSH content and PAL activity in tomato fruits. KIO_3_ also positively influenced GSH content in leaves while negatively affecting PAL and APX activities. Na_2_SeO_3_ favored GSH content and GPX activity in tomato fruits and leaves. Na_2_SeO_3_ negatively affected the antioxidant capacity of hydrophilic compounds by ABTS in fruits and leaves and favored hydrophilic compounds by DPPH in leaves. Seed imbibition based on KIO_3_ and Na_2_SeO_3_ is a method that is implemented in the tomato crop and presents interesting aspects that favor the nutraceutical quality of tomato fruits, which may contribute to increasing the intake of these minerals in humans through tomato consumption.

## 1. Introduction

Seed imbibition is considered a water-based technique that allows controlled hydration of the seed to trigger pregerminative metabolism but does not allow radicle emergence [1]. Seed imbibition is a common and effective process used to improve nutritional quality. The germination process is affected by external factors such as temperature, relative humidity, and light conditions, which can help or inhibit germination in relation to the nutritional reserve within the seed. During the germination process, some seed reserves are degraded and used for respiration and the synthesis of new cellular constituents for embryonic development, which causes changes in biochemical and nutritional characteristics [2].

The seed imbibition process consists of dipping the seeds in a water solution for some time, or imbibition time, allowing partial hydration without the radicle emerging. When a dry seed is kept in water, the germination process of non-dormant seeds occurs in three phases: imbibition, the lag phase, and protrusion of the radicle through the testa [3,4]. Seed priming is an effective method to improve seed physiological quality through uniformity and germination percentage [5]. It stimulates pre-germination metabolic processes, reduces the physical resistance of the endosperm during imbibition, repairs membranes, influences the development of immature embryos, and leaches inhibitors [3,6]. Seedlings from seed imbibition emerge faster, grow more vigorously, and tolerate adverse conditions (Table S1) related to certain physiological, biochemical, cellular, and molecular changes [7,8,9].

Treatments based on seed priming have been established as an effective method to improve the response of plants to biotic and abiotic stress conditions through the alteration of antioxidant metabolism [10]. This technique also stimulates the antioxidant system by improving the activity of antioxidant enzymes [11,12]. The use of iodine (I) and selenium (Se) in agriculture has become very relevant and important worldwide. At low concentrations, these elements present stimulatory effects and can favor the growth and development of crops and increase the content of secondary metabolites and antioxidant enzymes; however, at high concentrations, these elements can cause toxicity to plants.

Se plays an important role in the benefit of crop plants and is considered a biostimulant that promotes positive effects on the growth and development of crops. Se in the plants influences the accumulation of enzymes such as superoxide dismutases, catalases, ascorbate peroxidases, and glutathione reductases [13,14], which control the antioxidant metabolites, the production, and accumulation of reactive oxygen species (ROS), and activate the defense system [13]. Iodine and selenium are highly important in human health. The intake of these trace elements through food consumption is limited, which can cause malnutrition reflected in the abnormal development and growth of humans. These elements, required in small quantities, fulfill essential functions in the proper functioning of the thyroid gland (Table S2), which has recently been shown to be associated with carcinogenesis and its treatment in various cell lines, acting as an antioxidant and as an antiproliferative [15,16,17].

Tomato (*Solanum lycopersicum* L.) is a horticultural crop of worldwide importance due to its wide consumption as a processed byproduct and fresh presentation. This research aimed to evaluate the effect of seed priming based on I and Se on the nutraceutical quality of tomato fruits and leaves.

## 2. Materials and Methods

### 2.1. Crop Establishment

The tomato crop was established in a tunnel-type greenhouse with a plastic cover and natural ventilation in the Horticulture Department at the Universidad Autónoma Agraria Antonio Narro, in Saltillo Mexico (25°21′ NL, 101°01′ WL, altitude 1743 m). The average environmental conditions in the greenhouse during the crop cycle were: air temperature of 20 °C, relative humidity of 34%, solar radiation of 735 W m^−2^ and photosynthetically active radiation of 568 µmol m^−2^ s^−1^. Potassium iodate (KIO_3_) (99%, Sigma Aldrich, St. Louis, MO, USA) and sodium selenite (Na_2_SeO_3_) (99%, Sigma Aldrich, St. Louis, MO, USA) were used as I and Se species, respectively. Saladette-type CID F1 (Harris Moran^®^, Davis, CA, USA) tomato seeds were primed in KIO_3_ and Na_2_SeO_3_ solutions, and the seeds of the control treatment were primed in distilled water (Table 1) for a 24-h imbibition time (Figure S1). Then, the seeds were dried at room temperature for 48 h.

#### 2.1.1. Preparation of KIO_3_ and Na_2_SeO_3_ Treatments

A stock solution of potassium iodate (KIO_3_) at a concentration of 1000 ppm was prepared. A mass of 168.59 mg of KIO_3_ was gauged to 100 mL with distilled water. Dilutions of 2.5, 3.75, 5, and 6.25 mL of the stock solution were gauged to 25 mL with distilled water to obtain the treatments of 100, 150, 200, and 250 mg L^−1^, respectively.

A stock solution of sodium selenite (Na_2_SeO_3_) at a concentration of 1000 ppm was prepared. A mass of 21.89 mg of Na_2_SeO_3_ was gauged to 100 mL with distilled water. Dilutions of 12.5, 25, 50, and 75 µL of the stock solution were gauged to 25 mL with distilled water to obtain the treatments of 0.5, 1, 2, and 3 mg L^−1^, respectively.

#### 2.1.2. Sowing and Planting

After the seed priming process, seeds were sown in polystyrene trays with peat moss and perlite 1:1 (*v*/*v*) as substrates. Seedlings were planted 35 days after sowing in 10-L plastic containers with peat moss and perlite 1:1 (*v*/*v*) as substrate. Tomato plants were arranged in the greenhouse in a randomized complete block design with a factorial arrangement of two factors (KIO_3_ and Na_2_SeO_3_) and five levels (concentrations in mg L^−1^) (Table 1). Fertilization consisted of Steiner-type nutrient solutions [18], diluted in drip irrigation. The same substrate and fertigation conditions were used in the control and KIO_3_ and Na_2_SeO_3_ treatments to avoid another variation factor affecting the performance of the treatment.

#### 2.1.3. Sampling

Samples of the leaves and ripe fruits of tomato plants were obtained 120 days after planting. Leaf samples were collected from the leaf tissue of fully extended young leaves from 12 plants with four replications. Samples for biochemical analysis were collected from five ripe fruits per treatment, with a uniform color and size corresponding to stage six of ripening [19]. Samples were stored at −80 °C, lyophilized in a 2.5 L FreeZone Benchtop Free Dry System freeze-dryer (LABCONCO, Kansas, MO, USA), and ground to a fine powder.

### 2.2. Non-Enzymatic Compounds

#### 2.2.1. Vitamin C

The vitamin C content was determined by the 2,6-dichlorophenolindophenol titration method [20]. Here, 20 g of fresh fruit tissue was macerated by using a mortar and pestle with 10 mL of hydrochloric acid (2%), and 100 mL of distilled water was added and filtered with sterile gauze A 10-mL aliquot was taken and titrated with 2,6-dichlorophenolindophenol until a pinkish color was obtained (Equation (1)). The results were expressed in mg per 100 g of fresh tissue (mg 100 g^−1^ FW).

(1) $$\mathrm{Vitamin}\;C=\frac{\mathrm{mL}\;\mathrm{of}\;\text{2,6-dichlorophenolindophenol}\times 0.088\times \mathrm{total}\;\mathrm{volume}\times 100}{\mathrm{aliquot}\;\mathrm{volume}\times \mathrm{sample}\;\mathrm{weight}}$$

#### 2.2.2. Total Phenols

Total phenolic compounds were determined using the Folin–Ciocalteu method from the extraction with water:acetone [21], made by adding 50 µL of the extract, 200 µL folin–ciocalteu reagent, 500 µL Na_2_CO_3_, and 5 mL distilled water to a test tube. The mixture was vortexed for 30 s and placed in a water bath at 45 °C for 30 min. The absorbance of the mixture was read at a 750 nm wavelength. The results were expressed in mg of gallic acid equivalents per gram of dry tissue (mg GAE g^−1^ DW).

#### 2.2.3. Total Flavonoids

Flavonoids were determined by the Dowd method, adapted by Arvouet–Grand et al. [22]. It was made by adding 2 mL of the extract and 2 mL of AlCl_3_ (2%) methanolic solution to a test tube. The mixture was allowed to react for 20 min in the dark. The absorbance of the mixture was read at a 415 nm wavelength. The results were expressed in mg of quercetin equivalents per 100 g of dry tissue (mg QE 100 g^−1^ DW).

#### 2.2.4. Chlorophyll

Chlorophyll content was quantified using the method proposed by Munira et al. [23]. Here, 1 g of fresh plant material was homogenized, and then 5 mL acetone (90%) was added. Additionally, 10 mg of magnesium carbonate (to protect and stabilize chlorophylls) was added, 2 mL of the extract was centrifuged at 9408× *g* for 5 min at 4 °C, and the supernatant was extracted. Chlorophyll-*a* and chlorophyll-*b* were quantified by reading the absorbances at 663 and 645 nm wavelengths, respectively. The results were computed (Equations (2)–(4)) and expressed in µg per gram of fresh tissue (µg g^−1^ FW).

Chlorophyll-*a* = 3.64 × A_645_ + 25.38 × A_663_ (2)

Chlorophyll-*b* = 30.38 × A_645_ − 6.58 × A_663_ (3)

Total chlorophyll = 34.02 × A_645_ + 18.8 × A_663_ (4)

#### 2.2.5. Lycopene and β-carotene

Lycopene and β-carotene contents were determined according to Nagata and Yamashita [24]. Here, 100 mg of lyophilized tissue was mixed with 2 mL of hexane:acetone (3:2) solution. An aliquot was taken from the supernatant and the absorbances at 453, 505, 645, and 663 nm wavelengths were read. The results were computed (Equations (5) and (6)) and expressed in mg per 100 g of dry tissue (mg 100 g^−1^ DW).

Lycopene = −0.0806 × A_453_ + 0.372 × A_505_ + 0.204 × A_645_ − 0.0458 × A_663_ (5)

β-carotene = 0.452 × A_453_ − 0.304 × A_505_ − 1.22 × A_645_ + 0.216 × A_663_ (6)

### 2.3. Enzymatic Activity

#### 2.3.1. Extraction

Samples of leaves and ripe fruits of tomatoes were freeze-dried and macerated by using a mortar and pestle; 200 mg of dry tissue and 20 mg of polyvinyl pyrrolidone were added in a 2-mL centrifuge tube; 1.5 mL of phosphate buffer (0.1 M, pH 7–7.2) was added; and the mixture was subjected to sonication for 5 min, and then centrifuged in a Prism C2500 refrigerated microcentrifuge (Labnet International Inc., Edison, NJ, USA) at 14,700× *g* for 10 min at 4 °C. The supernatant was collected and filtered with a 0.45-mm-diameter nylon membrane. Finally, the supernatant was diluted with phosphate buffer (1:20). This dilution was used to analyze the absorbances of reduced glutathione (GSH), glutathione peroxidase (GPX), phenylalanine ammonium lyase (PAL), catalase (CAT), and ascorbate peroxidase (APX) in a GENESYS 10S UV-Vis Spectrum (Thermo Fisher Scientific, Inc., Waltham, MA, USA), as well as the antioxidant capacity of ABTS and DPPH radicals in a BK-EL10C Elisa microplate reader (BIOBASE, Jinan, Shandong, China) at the corresponding wavelengths.

#### 2.3.2. Reduced Glutathione (GSH)

GSH quantification was performed by the spectrophotometric technique [25]. It was made by adding 0.48 mL of the extract, 2.2 mL of Na_2_HPO_4_ (0.32 M), and 0.32 mL of DTNB (1 mM) to a test tube. The sample was allowed to react for 15 min at room temperature, and the absorbance was read at a 412 nm wavelength. The results were expressed in units per gram of total protein (U g^−1^ TP), where U is equal to mM of GSH equivalents per mL per minute of dry tissue (mM GSHE mL^−1^ min^−1^ DW).

#### 2.3.3. Glutathione Peroxidase (GPX) (QE 1.11.1.9)

GPX was determined using the Flohé and Günzler [26] method, adapted by Xue et al. [25]. It was performed by adding 0.2 mL of the extract, 0.5 mL of GSH (1 mM), and 0.2 mL of Na_2_HPO_4_ (0.067 M) to a test tube. The sample was placed in a water bath at 25 °C for 5 min, and 0.2 mL of H_2_O_2_ was added. The mixture was allowed to react for 10 min, and the reaction was stopped with 1 mL of trichloroacetic acid. The mixture was centrifuged in a C2500 refrigerated microcentrifuge (Labnet Prism^®^) at 846× *g* for 10 min at 4 °C. Then, 0.48 mL of the obtained mixture, 2.2 mL of Na_2_HPO_4_ (0.32 M), and 0.32 mL of 5.5 dithionis-2 nitro benzoic acid dye (1 mM) were added to a test tube. The mixture absorbance was read at a 412 nm wavelength. The results were expressed in units per gram of total protein (U g^−1^ TP), where U is equal to mM of GSH equivalents per mL per minute of dry tissue (mM GSHE mL^−1^ min^−1^ DW).

#### 2.3.4. Phenylalanine Ammonium Lyase (PAL) (QE 4.3.1.5)

PAL was determined according to Sykłowska–Baranek et al. [27]. It was performed by adding 0.1 mL of the extract and 0.9 mL of L-phenylalanine (6 mM) to a test tube. The mixture was incubated in a water bath for 30 min at 40 °C, and 0.25 mL of HCl (5 N) was added. The sample was placed in an ice bath, and 5 mL of distilled water was added. The mixture absorbance was read at a 290 nm wavelength. The results were expressed in units per 100 g of total protein (U 100 g^−1^ TP), where U is equal to μmol of trans-cinnamic acid equivalents per mL per minute of dry tissue (µmol TCAE mL^−1^ min^−1^ DW).

#### 2.3.5. Catalase (CAT) (QE 1.11.1.6)

CAT was determined by the spectrophotometric method [28], which is based on the quantification of the H_2_O_2_ oxidation rate by absorbance difference (T_0_ − T_1_). T_0_ was determined by adding 0.1 mL of the extract, 0.4 mL of H_2_SO_4_ (5%), and 1 mL of H_2_O_2_ (100 mM) to a test tube. T_1_ was computed by adding 0.1 mL of extract and 1 mL of H_2_O_2_ (100 mM), stirring for 1 min, and adding 0.4 mL of H_2_SO_4_ (5%). The mixture absorbance was read at a 270 nm wavelength. The results were expressed in units per gram of total protein (U g^−1^ TP), where U is equal to mM of H_2_O_2_ equivalents spent per mL per minute of dry tissue (mM H_2_O_2_E mL^−1^ min^−1^ DW).

#### 2.3.6. Ascorbate Peroxidase (APX) (EC 1.11.1.11)

APX quantification was performed according to the Nakano and Asada [29] method, which is based on the quantification of the H_2_O_2_ oxidation rate by absorbance difference (T_0_ − T_1_). T_0_ was determined by adding 0.1 mL of the extract, 0.5 mL of ascorbate (10 mg L^−1^), 0.4 mL of H_2_SO_4_ (5%) to stop the reaction, and 1 mL of H_2_O_2_ (100 mM) to a test tube. T_1_ was computed by adding 0.1 mL of extract and 1 mL of H_2_O_2_ (100 mM) to a test tube, stirring for 1 min, and adding 0.4 mL of H_2_SO_4_ (5%) to stop the reaction. The mixture absorbance was read at a 266 nm wavelength. The results were expressed in units per gram of total protein (U g^−1^ TP), where U is equal to μmol of ascorbate oxidized equivalents per mL per minute of dry tissue (μmol AOE mL^−1^ min^−1^ DW).

### 2.4. Antioxidant Capacity

#### 2.4.1. Antioxidant Capacity of Hydrophilic and Lipophilic Compounds by ABTS

Antioxidant activity by the ABTS radical (2,2′-azino-bis-3-ethylbenzothiazolin-6-sulfonic acid) was determined by the spectrophotometric method [30]. It is based on the discoloration of the ABTS radical cation. This radical was obtained from the reaction of ABTS (7 mM) with potassium persulfate (2.45 mM) (1:1) under dark conditions at 25 °C for 16 h. Subsequently, it was diluted with ethanol (20%) to obtain an absorbance of 0.7 ± 0.01 at a 750 nm wavelength.

The antioxidant capacity of hydrophilic compounds by ABTS was determined by sample extraction performed with phosphate buffer, where 5 μL of extract and 245 μL of the dilution of ABTS radicals (7 mM) were placed in a microplate and allowed to react for 7 min in the dark. The absorbance of the extract was measured at a 750 nm wavelength. 

The antioxidant capacity of lipophilic compounds as determined by ABTS was calculated by sample extraction performed with a hexane:acetone solution (3:2). Both the antioxidant capacity results of hydrophilic and lipophilic compounds by ABTS were expressed in µmol of Trolox equivalents per gram of dry tissue (µmol TE g^−1^ DW).

#### 2.4.2. Antioxidant Capacity of Hydrophilic Compounds by DPPH

Antioxidant capacity by DPPH radical (2,2-Diphenyl-1-picrylhydrazyl) was performed according to Brand–Williams et al. [31]. The stock solution was prepared by mixing 2.5 mg of the DPPH radical with 100 mL of methanol. The absorbance of the solution was adjusted to 0.7 ± 0.02 at a 515 nm wavelength. 

The antioxidant capacity of hydrophilic compounds by DPPH was determined as follows: 6 μL of the obtained extract was taken with phosphate buffer, and 234 μL of the DPPH radical was added. The decrease in absorbance of the extract after 30 min was measured at a 515 nm wavelength. The antioxidant capacity results of hydrophilic compounds by DPPH were expressed in µmol of Trolox equivalents per gram of dry tissue (µmol TE g^−1^ DW). 

#### 2.4.3. Statistical Analyses

The results were analyzed by analysis of variance to determine the variables that presented a significant statistical difference (*p* ≤ 0.05) so that the variables with significant effects were submitted to comparison means tests by Tukey (*p* ≤ 0.05) using the statistical software InfoStat^®^ 2020e.

## 3. Results

### 3.1. Non-Enzymatic Compounds in Tomato Fruits by KIO_3_ and Na_2_SeO_3_ Interactions

The lycopene and β-carotene contents of tomato fruits were significantly modified by KIO_3_ and Na_2_SeO_3_ interactions. The lycopene content increased 110.6% in the 200–2 mg L^−1^ interaction and decreased 77.3% in the 200–3 mg L^−1^ interaction in relation to the control treatment. The β-carotene content increased 157.1% in the 200–1 mg L^−1^ interaction and decreased 90.5% in the 0–0.5 mg L^−1^ interaction in relation to the control treatment (0–0 mg L^−1^ interaction).

Phenol and flavonoid contents in tomato fruits significantly decreased in response to KIO_3_ and Na_2_SeO_3_ interactions. The phenol content increased by 9.7% in the 200–1 mg L^−1^ interaction and decreased by 32.3% in the 150–1 mg L^−1^ interaction in relation to the control treatment. Flavonoid content increased by 13.3% in the 250–1 mg L^−1^ interaction and decreased by 35% in the 200–3 mg L^−1^ interaction in relation to the control treatment.

The vitamin C content in tomato fruits was non-significantly influenced by KIO_3_ and Na_2_SeO_3_ interactions (Table 2).

### 3.2. Non-Enzymatic Compounds in Tomato Fruits by KIO_3_ and Na_2_SeO_3_ Factors

**Figure 1:**
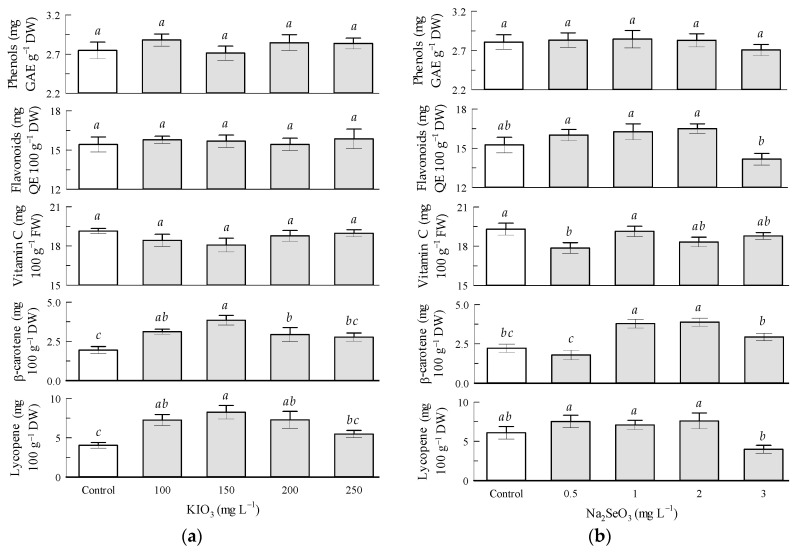
Non-enzymatic antioxidant compounds in tomato fruits: (**a**) Seed priming based on KIO_3_; (**b**) Seed priming based on Na_2_SeO_3_. Different letters indicate significant differences between treatments (Tukey HSD, *p* ≤ 0.05). n = 4.

Regarding the potassium iodate factor in the tomato fruits, the vitamin C content significantly decreased by 5.2% with KIO_3_ in the 150 mg L^−1^ treatment in relation to the control treatment. Phenol and flavonoid contents were not influenced by KIO_3_. Lycopene and β-carotene contents significantly increased with all KIO_3_ treatments, where the 150 mg L^−1^ dose allowed reaching increments of 105 and 100% on lycopene and β-carotene contents, respectively, in relation to the control treatment, which contrasts with the results of vitamin C (Figure 1a).

Regarding the sodium selenite factor in the tomato fruits, the vitamin C content significantly increased by 9% with Na_2_SeO_3_ in the 1 mg L^−1^ treatment in relation to the 0.5 mg L^−1^ treatment. The phenol content was not influenced by Na_2_SeO_3_. Flavonoid content increased by 8.6% with Na_2_SeO_3_ in the 2 mg L^−1^ treatment and decreased by 7.2% in the 3 mg L^−1^ doses in relation to the control treatment. The lycopene content increased by 25% with Na_2_SeO_3_ in the 0.5 and 2 mg L^−1^ treatments and decreased by 35% in the 3 mg L^−1^ treatment, in relation to the control treatment. The β-carotene content increased with Na_2_SeO_3_ at 1–3 mg L^−1^, reaching the highest increase by 72.7% in the 2 mg L^−1^ treatment in relation to the control treatment. The Na_2_SeO_3_ treatment at 2 mg L^−1^ presented the best performance because it resulted in the highest increases in lycopene, β-carotene, and flavonoid contents in tomato fruits in relation to the corresponding control treatment (Figure 1b).

### 3.3. Non-Enzymatic Compounds in Tomato Leaves by KIO_3_ and Na_2_SeO_3_ Interactions

The phenol content was significantly influenced by KIO_3_ and Na_2_SeO_3_ interactions, that is, increased by 31.6% in the 100–0.5 mg L^−1^ interaction and decreased by 28.4% in the 0–0.5 mg L^−1^ interaction in relation to the control treatment (0–0 mg L^−1^ interaction). The flavonoid content significantly decreased by 21.4% in the 100–3 mg L^−1^ interaction in relation to the control treatment. Chlorophyll and β-carotene were non-significantly influenced by KIO_3_ and Na_2_SeO_3_ interactions in relation to the respective control treatments.

Chlorophyll-*a* and total chlorophyll significantly increased by 3.2% and 2.4%, respectively, in the 100–0 mg L^−1^ interaction in relation to the 0–3 mg L^−1^ interaction, that is, removing the KIO_3_ and raising the Na_2_SeO_3_ doses (Table 3).

### 3.4. Non-Enzymatic Compounds in Tomato Leaves by KIO_3_ and Na_2_SeO_3_ Factors

**Figure 2:**
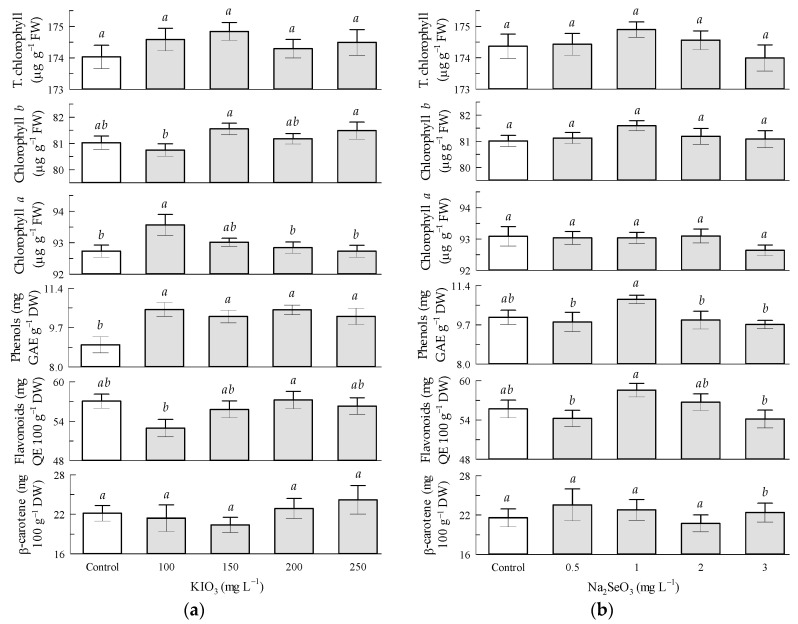
Non-enzymatic antioxidant compounds in tomato leaves: (**a**) Seed priming based on KIO_3_; (**b**) Seed priming based on Na_2_SeO_3_. Different letters indicate significant differences between treatments (Tukey HSD, *p* ≤ 0.05). n = 4.

Regarding the potassium iodate factor in the tomato leaves, the phenol content significantly increased with all KIO_3_ treatments, where the 100 and 200 mg L^−1^ treatments reached increments of 16.9% in relation to the control treatment. The chlorophyll-*a* content increased by 0.9% with KIO_3_ in the 100 mg L^−1^ treatment in relation to the control treatment. Flavonoid, chlorophyll-*b*, and β-carotene contents were not influenced by KIO_3_ (Figure 2a).

Regarding the sodium selenite factor in the tomato leaves, all the non-enzymatic antioxidant compounds were not significantly influenced by Na_2_SeO_3_ (Figure 2b).

### 3.5. Enzymatic Activity in Tomato Fruits by KIO_3_ and Na_2_SeO_3_ Interactions

Regarding the tomato fruits, the (GSH) content and the PAL enzymatic activity were significantly increased by KIO_3_ and Na_2_SeO_3_ interactions: GSH by 35% in the 150–0.5 mg L^−1^ interaction and PAL by 441.7% in the 100–1 mg L^−1^ interaction in relation to the respective control treatments (0–0 mg L^−1^ interaction).

On the other hand, the enzymatic activities of GPX, CAT, and APX were not significantly influenced by KIO_3_ and Na_2_SeO_3_ interactions in relation to the respective control treatments.

Lower and higher changes in GPX values were −30.6 and 238.9% in the 100–2 and 0–3 mg L^−1^ interactions, respectively, in relation to the corresponding control treatments. Lower and higher changes in CAT values were −14.8 and 70.4% in the 0–3 and 100–3 mg L^−1^ interactions, respectively, in relation to the corresponding control treatments. Lower and higher changes in APX values were −46.4 and 21.4% in the 150–1 and 100–2 mg L^−1^ interactions, respectively, in relation to the corresponding control treatments (Table 4).

### 3.6. Enzymatic Activity in Tomato Fruits by KIO_3_ and Na_2_SeO_3_ Factors

**Figure 3:**
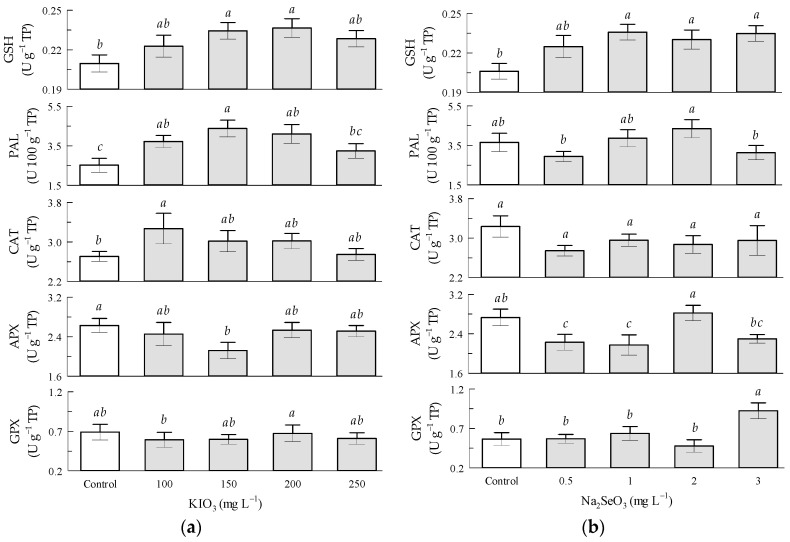
Enzymatic antioxidant compounds in tomato fruits: (**a**) Seed priming based on KIO_3_; (**b**) Seed priming based on Na_2_SeO_3_. Different letters indicate significant differences between treatments (Tukey HSD, *p* ≤ 0.05). n = 4.

Regarding the potassium iodate factor in the tomato fruits, the KIO_3_ treatments at 150 and 200 mg L^−1^ significantly increased the GSH content by 9.5 and 14.3%, and the PAL enzymatic activity by 72 and 64%, respectively, in relation to the corresponding control treatments. GPX, CAT, and APX enzymatic activities were not significantly influenced by KIO_3_ (Figure 3a).

Regarding the sodium selenite factor in the tomato fruits, the GSH content was significantly increased by Na_2_SeO_3_ by 14.3% and 9.5% in the 1 and 2–3 mg L^−1^ treatments, respectively, in relation to the corresponding control treatments. GPX enzymatic activity significantly increased by 64.3% by Na_2_SeO_3_ in the 3 mg L^−1^ treatment in relation to the control treatment (Figure S2). PAL, CAT, and APX enzymatic activities were not significantly influenced by Na_2_SeO_3_ in relation to the respective control treatments. APX enzymatic activity significantly increased by 33.3% by Na_2_SeO_3_ in the 2 mg L^−1^ treatment in relation to the 1 mg L^−1^ treatment (Figure 3b).

### 3.7. Enzymatic Activity in Tomato Leaves by KIO_3_ and Na_2_SeO_3_ Interactions

GSH content and enzymatic activity of GPX in tomato leaves were significantly increased by KIO_3_ and Na_2_SeO_3_ interactions; GSH by 27.3% in the 200–0 mg L^−1^ interaction and GPX by 80% in the 150–0.5 mg L^−1^ interaction, in relation to the respective control treatments (0–0 mg L^−1^ interaction). The enzymatic activities of PAL, CAT, and APX were not significantly modified by KIO_3_ and Na_2_SeO_3_ interactions.

Higher enzymatic activities occurred for PAL in the 0–0.5 mg L^−1^ interaction, for CAT in the 250–0.5 mg L^−1^ interaction, and for APX in the 0–2 mg L^−1^ interaction (Table 5).

### 3.8. Enzymatic Activity in Tomato Leaves by KIO_3_ and Na_2_SeO_3_ Factors

**Figure 4:**
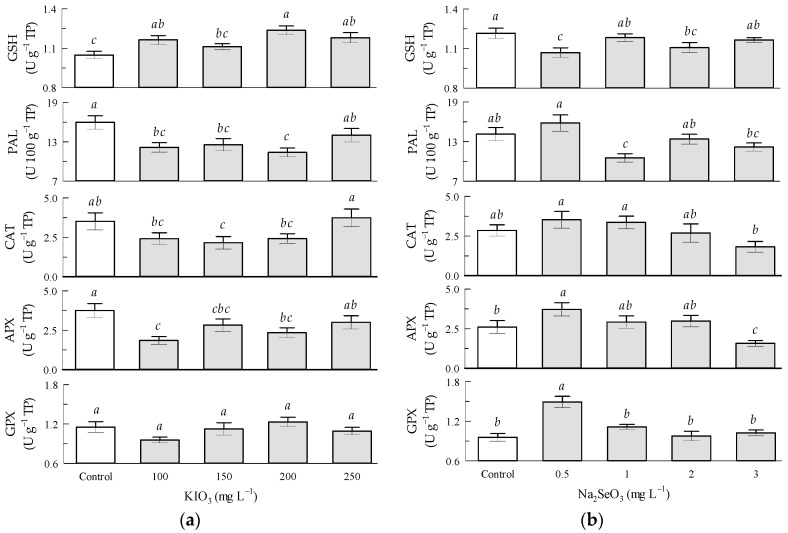
Enzymatic antioxidant compounds in tomato leaves: (**a**) Seed priming based on KIO_3_; (**b**) Seed priming based on Na_2_SeO_3_. Different letters indicate significant differences between treatments (Tukey HSD, *p* ≤ 0.05). n = 4.

Regarding the potassium iodate factor in the tomato leaves, by increasing the KIO_3_ concentration from 100 to 200 mg L^−1^, the GSH content significantly increased from 10 to 20%, the enzymatic activity of PAL decreased from 23.9 to 28.9%, and the enzymatic activity of APX decreased from 51.4 to 37.8%. The enzymatic activities of CAT and APX were not significantly influenced by KIO_3_ (Figure 4a).

Regarding the sodium selenite factor in the tomato leaves, the GSH content significantly decreased by Na_2_SeO_3_ by 16.7 and 8.3% in the 0.5 and 2 mg L^−1^ treatments, respectively, in relation to the control treatment. The enzymatic activity of GPX significantly increased by 55.6% with Na_2_SeO_3_ in the 0.5 mg L^−1^ treatment in relation to the control treatment. The enzymatic activities of PAL, CAT, and APX were not significantly influenced by Na_2_SeO_3_ (Figure 4b).

### 3.9. Antioxidant Capacity in Tomato Fruits and Leaves by KIO_3_ and Na_2_SeO_3_ Interactions

Regarding the tomato fruits, the antioxidant capacity of hydrophilic compounds by the ABTS radical significantly decreased by 64.1% in the 200–2 mg L^−1^ (KIO_3_-Na_2_SeO_3_) interaction in relation to the control treatment (0–0 mg L^−1^ interaction). The antioxidant capacity of lipophilic compounds by ABTS and hydrophilic compounds by DPPH radicals was not significantly influenced by KIO_3_ and Na_2_SeO_3_ interactions. Higher values of antioxidant capacity of hydrophilic compounds by DPPH occurred in the 0–0.5 mg L^−1^ interaction (Table 6).

Regarding the tomato leaves, the antioxidant capacity of hydrophilic compounds by DPPH radical significantly increased by 187% in the 200–2 mg L^−1^ interaction, in relation to the control treatment. The antioxidant capacity of hydrophilic and lipophilic compounds by the ABTS radicals was not significantly influenced by KIO_3_ and Na_2_SeO_3_ interactions. Higher values of antioxidant capacity of hydrophilic compounds by ABTS occurred in the 150–3 mg L^−1^ interaction and for lipophilic compounds by ABTS in the 200–0.5 mg L^−1^ interaction (Table 6).

### 3.10. Antioxidant Capacity in Tomato Fruits and Leaves by KIO_3_ and Na_2_SeO_3_ Factors

**Figure 5:**
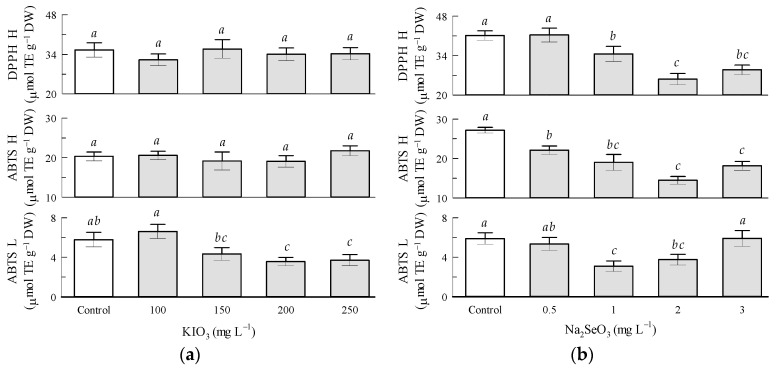
Antioxidant capacity of tomato fruits: (**a**) Seed priming based on KIO_3_; (**b**) Seed priming based on Na_2_SeO_3_. Different letters indicate significant differences between treatments (Tukey HSD, *p* ≤ 0.05). n = 4.

**Figure 6:**
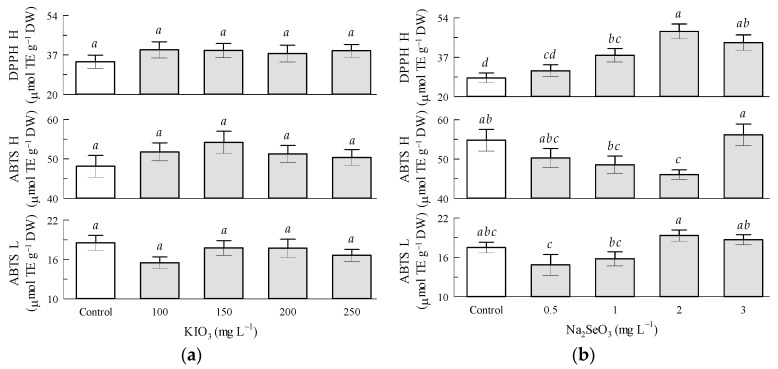
Antioxidant capacity of tomato leaves: (**a**) Seed priming based on KIO_3_; (**b**) Seed priming based on Na_2_SeO_3_. Different letters indicate significant differences between treatments (Tukey HSD, *p* ≤ 0.05). n = 4.

Regarding the potassium iodate factor, the antioxidant capacity of hydrophilic compounds by ABTS and by DPPH and of lipophilic compounds by ABTS was not significantly influenced by KIO_3_ in tomato fruits (Figure 5a) and leaves (Figure 6a). The antioxidant capacity of lipophilic compounds by ABTS in tomato fruits increased 88.6% with KIO_3_ in the 100 mg L^−1^ treatment in relation to the 200 mg L^−1^ treatment (Figure 5a).

Regarding the sodium selenite factor in the tomato fruits, the antioxidant capacity of hydrophilic compounds by ABTS and by DPPH was negatively affected by Na_2_SeO_3_, where the 2 mg L^−1^ treatment presented higher inhibition of these parameters by 46.9 and 37.7%, respectively (Figure 5b).

Regarding the sodium selenite factor in the tomato leaves, the antioxidant capacity of hydrophilic compounds by DPPH significantly increased by Na_2_SeO_3_ 35.4%, 71.8%, and 54.6% in the 1, 2, and 3 mg L^−1^ treatments, respectively. The antioxidant capacity of hydrophilic compounds by ABTS significantly decreased by 15.9% by Na_2_SeO_3_ in the 2 mg L^−1^ treatment in relation to the control treatment. The antioxidant capacity of lipophilic compounds by ABTS was not significantly influenced by Na_2_SeO_3_ in tomato fruits and leaves (Figure 6b).

## 4. Discussion

Implementation of techniques such as seed imbibition is an effective method to improve the response of plants to biotic and abiotic stress conditions (Table S1) through the alteration of antioxidant metabolism [10]. Important findings of seed imbibition are reported, such as the antioxidant response of broccoli influenced by selenium [32], the salt stress tolerance of strawberries influenced by iodine species [33], and the functional effects of selenium in crucifers [34].

Plants can absorb different chemical elements from the soil or the nutrient solution, whether these elements are beneficial or toxic [35]. Selenium can be absorbed through the roots in the form of selenate (SeO_4_^2−^), selenite (SeO_3_^2−^), and organic Se compounds such as selenocysteine (SeCys) and selenomethionine (SeMet) (Table S2), but selenides or elemental Se cannot be absorbed [36]. The use of selenium in plants has been reported to have positive effects on glutathione content, because selenium increases sulfur (S) receptors and consequently increases the absorption of both elements, favoring the synthesis of secondary metabolites [37].

Plant cells produce free oxygen and its derivatives, such as reactive oxygen species (ROS), which are used as signaling molecules in plants in signal translation in response to environmental conditions; this triggers antioxidant defense mechanisms. In this context, it has been shown that the optimal addition of iodine (Figure S3) and selenium presents an alteration in the ROS production system [38,39], where the enzymatic defense systems, such as catalase, peroxidase, superoxide dismutase, glutathione peroxidase, and ascorbate peroxidase, and non-enzymatic antioxidants, such as glutathione, ascorbate, tocopherols, and phenolic compounds, are activated (Figure S4) to reduce the excessive ROS production [40]. For its part, the SOD enzyme dismutases the O_2_^−^ into hydrogen peroxide H_2_O_2_ and molecular oxygen O_2_, and later the catalase (CAT) degrades the H_2_O_2_ into oxygen and water, while the ascorbate peroxidase uses the ascorbic acid as a donor to stimulate the degradation of H_2_O_2_, and the reduced glutathione is responsible for the production of ascorbic acid [41]. Glutathione reductase catalyzes the regeneration of reduced glutathione (GSH) from glutathione disulfide (GSSG) with NADPH as the reducing agent. GSH eliminates H_2_O_2_ by non-enzymatic reaction with O_2_^−^ and OH^−^, likewise, GSH has the ability to replenish ascorbic acid through the ascorbate-glutathione cycle, which is of great importance for the antioxidant system [42].

### 4.1. Non-Enzymatic Compounds

In this research, the use of iodine and selenium concentrations applied to tomato crops by seed priming presented increases in the content of flavonoids, lycopene, and carotene in tomato fruits (Figure S2). These results agree with those reported by Gaucin–Delgado et al. [43], who applied 2 mg L^−1^ Na_2_SeO_4_ in the nutrient solution, presenting an increase in the content of phenols in the tomato crop. Likewise, Cunha et al. [44] and Ishtiaq et al. [45] indicated that the use of selenium presents an increase in chlorophyll, carotenes, and phenolic compounds when using concentrations of 7.5 and 15 µg kg^−1^, while Sabatino et al. [46] indicated that the use of concentrations of 2 and 4 µmol of SeO_2_ presented a higher content in carotenes in relation to the control. Phenol and vitamin C contents in tomato fruits did not present significant effects between treatments, which are similar to those reported by Smoleń et al. [47], who indicated that the use of KIO_3_ in conjunction with Na_2_SeO_3_ at concentrations of 30 and 8.5 µg dm^−3^, respectively, did not present a significant effect in relation to the control.

The use of KIO_3_ influenced an increase in the phenol and chlorophyll-*a* contents; however, the Na_2_SeO_3_ treatments did not significantly modify these parameters in the leaves in relation to the control. Similar results were reported by Jerse et al. [48], who mentioned that the use of Na_2_SeO_3_ at 10 mg L^−1^ in conjunction with KIO_3_ at concentrations of 1000 mg L^−1^ did not present an effect on photosynthetic compounds, likewise indicating that the use of iodine concentrations and selenium separately reduced the dry matter content. However, when both elements interacted, there was a higher biomass content, which is similar to that reported by Smoleń et al. [49], who found that the separate use of selenium and vanadium promotes iodine uptake in plants.

Wang et al. [50] indicated that imbibition treatments present an increase in the content of polyphenols in the rye, which is attributed to the synthesis or activation of a variety of hydrolytic enzymes, causing different alterations in the structure or the synthesis of new compounds with high bioactivity and nutritional value. On the other hand, Vicas et al. [51] indicated that the use of selenium nanoparticles did not affect the phenol content of the broccoli crop. Likewise, Islam et al. [52] indicated that at a higher concentration of Na_2_SeO_3_, the phenol content begins to decrease. In the same way, Shohag et al. [53] indicated that the imbibed soybean and bean seeds presented a decrease in the phenol content in seeds and sprouts. This decrease in the phenol content is attributed to the imbibition time (Figure S1) since it is considered that the longer the imbibition time, the greater the water absorption, which presents a dilution effect.

Jerše et al. [48] indicated that the use of iodine and selenium in the imbibition of seeds is a viable method because the enrichment of the pea shoots was achieved with the use of both elements; however, the uptake depends on the shape and/or combinations of the elements. The same effect was reported by Deng et al. [54] and Radawiec et al. [55], who indicated that in osmoconditioning treatments with iron, copper, manganese, zinc, selenium, and iodine in soybean and wheat seeds, they present an increase in the speed of germination and accumulation of these compounds in the shoots, for which they define seed imbibition treatments as a simple and highly efficient technique to increase the content of organic mineral elements in sprouts.

### 4.2. Enzymatic Activity

Regarding the enzymatic activity, the use of KIO_3_ presented an improvement in the GSH content and a greater enzymatic activity in PAL (Figure S3); similar results are reported by Blasco et al. [56], who indicated that the use of iodide (I^−^) and iodate (IO_3_^−^) in lettuce plants presents an increase in antioxidant enzymes. On the other hand, Na_2_SeO_3_ presented a higher GSH and GPX content (Figure S2); similar results were reported by Zhu et al. [13] and Rady et al. [57], where the use of selenium concentrations favors the increase in the GSH and GPX contents in the tomato crop. The increase in the GHS content is beneficial since high concentrations are needed to overcome oxidative stress in chloroplasts and other organelles [58].

Cao et al. [59], Diao et al. [60], and Kumar et al. [10] indicated that the use of Na_2_SeO_3_ increases the enzymatic activity compared to the control, presenting an increase in GPX, CAT, and APX, while Nawaz et al. [61] indicated that the enzymatic activity of CAT and APX is increased in seed imbibition treatments with Se. Hu et al. [62] indicated that the use of selenium in the seed imbibition solution presents an increase in the α-amylase content, an increase in the sugar content, and an increase in the enzymatic activity of superoxide dismutase (SOD), glutathione peroxidase (GPX), and catalase (CAT), in addition to presenting a higher total chlorophyll content and Se content in the seedlings; however, this depends on the imbibition time of the seeds. Nawaz et al. [61] indicated that using selenium and exogenous zinc in seed’s imbibition increases germination index, vigor, and enzymatic antioxidants such as catalase, guaiacol peroxidase, superoxide dismutase, and ascorbate peroxidase.

### 4.3. Antioxidant Capacity

Regarding the antioxidant capacity, there was a positive response when using Na_2_SeO_3_ in the leaves by DPPH of hydrophilic compounds, while Fuentes et al. [63], Medrano Macías et al. [33], and Sarrou et al. [64] indicated that the use of KIO_3_ does not affect the antioxidant capacity in strawberry and tomato crops.

## 5. Conclusions

The application of potassium iodate and sodium selenite in tomato crops by seed imbibition treatments influenced significant changes in non-enzymatic antioxidant compounds, such as phenols, lycopene, β-carotene, and reduced glutathione, as well as on enzymes, that is, phenylalanine ammonium lyase, catalase, and ascorbate peroxidase, both in tomato leaves and fruits; however, the same treatments influenced not significant changes in the antioxidant capacity. Seed priming based on trace elements is a useful and simple technique to perform in agricultural and horticultural production systems. Although this method does not present inconvenience due to the low concentrations of trace elements required, it is necessary to carry out more studies to establish the optimal concentrations according to the crop and the form of application, which allow for improvement of the desired indicators, such as the antioxidant compound pool of the edible organs of the plants, understand the balance and pathway of trace elements in the plant, and the benefits of biofortification of the fruits.

## Figures and Tables

**Table 1:** Priming treatments in tomato seeds based on I and Se.

Variation Factor	Concentration (mg L^−1^)
KIO_3_	0, 100, 150, 200, 250
Na_2_SeO_3_	0, 0.5, 1, 2, 3

25 treatments (5^2^ factorial), n = 4 replications, 100 experimental units.

**Table 2:** Effect of seed priming based on KIO_3_ and Na_2_SeO_3_ interactions on the non-enzymatic antioxidant compounds in tomato fruits.

Na_2_SeO_3_	KIO_3_	Vitamin C	Phenols	Flavonoids	Lycopene	β-carotene
(mg L^−1^)	(mg L^−1^)	(mg 100 g^−1^ FW)	(mg GAE g^−1^ DW)	(mg QE 100 g^−1^ DW)	(mg 100 g^−1^ DW)	(mg 100 g^−1^ DW)
0	0	18.8 *abcdef*	3.1 *ab*	18.0 *ab*	6.6 *cd*	2.1 *efg*
0	100	17.6 *bcdef*	2.9 *abc*	17.2 *abc*	12.4 *b*	3.8 *abcde*
0	150	21.5 *a*	2.7 *abc*	12.6 *fghi*	4.0 *efg*	1.9 *fgh*
0	200	20.5 *abc*	2.6 *abc*	15.8 *bcdef*	4.3 *efg*	1.6 *fgh*
0	250	18.0 *bcdef*	2.6 *abc*	12.4 *hi*	2.9 *gh*	1.5 *fgh*
0.5	0	19.2 *abcdef*	2.7 *abc*	13.4 *defghi*	4.3 *efg*	0.2 *h*
0.5	100	16.2 *f*	3.0 *abc*	15.4 *bcdefgh*	6.6 *cd*	2.1 *efg*
0.5	150	16.5 *ef*	2.6 *abc*	17.3 *abc*	13.8 *a*	3.9 *abcd*
0.5	200	17.9 *bcdef*	2.8 *abc*	15.7 *bcdefg*	4.9 *ef*	0.8 *gh*
0.5	250	19.5 *abcde*	2.9 *abc*	18.0 *ab*	7.8 *c*	1.7 *fgh*
1	0	19.1 *abcdef*	2.8 *abc*	12.9 *efghi*	3.8 *fg*	2.0 *fg*
1	100	20.6 *ab*	2.7 *abc*	15.1 *bcdefgh*	6.4 *cd*	3.0 *cdef*
1	150	17.1 *def*	2.1 *c*	15.9 *bcde*	6.6 *cd*	4.2 *abc*
1	200	19.8 *abcd*	3.4 *a*	16.8 *bc*	11.6 *b*	5.4 *a*
1	250	19.07 *abcdef*	3.0 *abc*	20.4 *a*	6.9 *c*	4.1 *abcd*
2	0	19.3 *abcde*	2.3 *bc*	17.2 *abc*	3.0 *gh*	2.6 *cdef*
2	100	18.2 *bcdef*	2.7 *abc*	14.8 *cdefghi*	3.2 *gh*	2.7 *cdef*
2	150	18 *bcdef*	3.0 *ab*	17.5 *abc*	11.2 *b*	5.2 *ab*
2	200	16.8 *def*	3.0 *abc*	17.1 *bc*	13.9 *a*	4.9 *ab*
2	250	19.3 *abcdef*	2.9 *abc*	15.8 *bcdef*	6.4 *cd*	3.7 *be*
3	0	19.2 *abcdef*	2.6 *abc*	15.3 *bcdefgh*	2.3 *hi*	2.5 *def*
3	100	19.5 *abcde*	2.9 *abc*	16.2 *bcd*	7.5 *c*	3.8 *abcde*
3	150	17.4 *cdef*	2.8 *abc*	14.9 *bcdefgh*	5.4 *de*	3.8 *abcd*
3	200	18.8 *abcdef*	2.3 *bc*	11.7 *i*	1.5 *i*	1.7 *fgh*
3	250	18.9 *abcdef*	2.7 *abc*	12.5 *ghi*	3.1 *gh*	2.6 *cdef*

Different letters within the columns indicate significant differences between the treatment interactions (Tukey HSD, *p* ≤ 0.05). n = 4.

**Table 3:** Effect of seed priming based on KIO_3_ and Na_2_SeO_3_ interactions on the non-enzymatic antioxidant compounds in tomato leaves.

Na_2_SeO_3_	KIO_3_	Phenols	Flavonoids	Chlorophyll *a*	Chlorophyll *b*	Total chl.	β-carotene
(mg L^−1^)	(mg L^−1^)	(mg GAE g^−1^ DW)	(mg QE 100 g^−1^ DW)	(µg g^−1^ FW)	(µg g^−1^ FW)	(µg g^−1^ FW)	(mg 100 g^−1^ DW)
0	0	9.5 *cdef*	60.4 *ab*	93.0 *abc*	81.3 *a*	174.7 *ab*	0.22 *a*
0	100	10.3 *abcde*	58.2 *abc*	94.4 *a*	81.0 *a*	175.8 *a*	0.15 *a*
0	150	10.5 *abcde*	52.8 *abc*	93.1 *abc*	81.3 *a*	174.7 *ab*	0.22 *a*
0	200	11.1 *abcd*	58.1 *abc*	92.8 *abc*	80.8 *a*	174.0 *ab*	0.25 *a*
0	250	8.5 *efg*	48.8 *abc*	91.9 *bc*	80.3 *a*	172.5 *ab*	0.23 *a*
0.5	0	6.8 *g*	51.4 *abc*	92.7 *abc*	80.6 *a*	173.6 *ab*	0.23 *a*
0.5	100	12.5 *a*	48.4 *bc*	94.0 *ab*	81.0 *a*	175.4 *a*	0.24 *a*
0.5	150	9.16 *efg*	55.4 *abc*	92.9 *abc*	81.3 *a*	174.5 *ab*	0.19 *a*
0.5	200	11.13 *bcd*	58.9 *abc*	92.3 *abc*	81.1 *a*	173.7 *ab*	0.24 *a*
0.5	250	9.4 *def*	56.8 *abc*	93.0 *abc*	81.4 *a*	174.7 *ab*	0.28 *a*
1	0	10.7 *abcde*	58.1 *abc*	92.9 *abc*	81.4 *a*	174.6 *ab*	0.23 *a*
1	100	10.1 *abcde*	54.2 *abc*	93.2 *abc*	80.6 *a*	174.1 *ab*	0.24 *a*
1	150	10.7 *abcde*	59.6 *abc*	92.8 *abc*	81.8 *a*	175.0 *ab*	0.21 *a*
1	200	10.5 *abcde*	61.0 *ab*	93.5 *abc*	81.8 *a*	175.7 *a*	0.21 *a*
1	250	11.8 *abc*	59.6 *abc*	92.5 *abc*	82.1 *a*	174.9 *ab*	0.25 *a*
2	0	7.6 *fg*	58.1 *abc*	93.3 *abc*	81.8 *a*	175.4 *a*	0.19 *a*
2	100	9.4 *cdef*	56.3 *abc*	93.6 *abc*	80.1 *a*	174.0 *ab*	0.18 *a*
2	150	11.0 *abcd*	50.4 *abc*	92.9 *abc*	81.0 *a*	174.1 *ab*	0.20 *a*
2	200	9.3 *def*	57.2 *abc*	92.9 *abc*	81.3 *a*	174.6 *ab*	0.21 *a*
2	250	12.1 *ab*	61.4 *a*	92.6 *abc*	81.6 *a*	174.5 *ab*	0.27 *a*
3	0	10.0 *bcdef*	57.1 *abc*	91.5 *c*	79.8 *a*	171.6 *b*	0.24 *a*
3	100	9.9 *bcdef*	47.5 *c*	92.4 *abc*	80.8 *a*	173.5 *ab*	0.26 *a*
3	150	9.3 *def*	60.7 *ab*	93.2 *abc*	82.2 *a*	175.7 *a*	0.20 *a*
3	200	10.2 *abcde*	50.7 *abc*	92.4 *abc*	80.6 *a*	173.3 *ab*	0.23 *a*
3	250	9.0 *defg*	54.7 *abc*	93.5 *abc*	81.9 *a*	175.6 *a*	0.18 *a*

Different letters within the columns indicate significant differences between the treatment interactions (Tukey HSD, *p* ≤ 0.05). n = 4.

**Table 4:** Effect of seed priming based on KIO_3_ and Na_2_SeO_3_ interactions on the enzymatic activity in tomato fruits.

Na_2_SeO_3_	KIO_3_	GSH	GPX	PAL	CAT	APX
(mg L^−1^)	(mg L^−1^)	(U g^−1^ TP)	(U g^−1^ TP)	(U 100 g^−1^ TP)	(U g^−1^ TP)	(U g^−1^ TP)
0	0	0.20 *bc*	0.36 *ab*	1.2 *b*	2.7 *ab*	2.8 *ab*
0	100	0.22 *abc*	0.59 *ab*	4.1 *ab*	2.9 *ab*	2.3 *ab*
0	150	0.21 *abc*	0.54 *ab*	4.4 *ab*	4.0 *ab*	2.7 *ab*
0	200	0.20 *abc*	0.77 *ab*	4.7 *ab*	3.1 *ab*	2.9 *ab*
0	250	0.20 *abc*	0.57 *ab*	3.7 *ab*	3.2 *ab*	2.7 *ab*
0.5	0	0.20 *bc*	0.47 *ab*	3.0 *ab*	2.6 *ab*	3.0 *ab*
0.5	100	0.20 *abc*	0.48 *ab*	3.6 *ab*	2.6 *ab*	1.9 *ab*
0.5	150	0.27 *a*	0.71 *ab*	3.8 *ab*	2.9 *ab*	1.6 *ab*
0.5	200	0.25 *abc*	0.72 *ab*	2.3 *ab*	3.0 *ab*	2.2 *ab*
0.5	250	0.21 *abc*	0.46 *ab*	1.7 *b*	2.5 *ab*	2.3 *ab*
1	0	0.20 *abc*	0.73 *ab*	3.0 *ab*	3.0 *ab*	2.0 *ab*
1	100	0.25 *abc*	0.43 *ab*	2.3 *ab*	2.8 *ab*	2.4 *ab*
1	150	0.22 *abc*	0.72 *ab*	4.1 *ab*	3.0 *ab*	1.5 *b*
1	200	0.26 *a*	0.70 *ab*	6.5 *a*	3.4 *ab*	2.4 *ab*
1	250	0.25 *abc*	0.60 *ab*	3.2 *ab*	2.4 *ab*	2.3 *ab*
2	0	0.21 *abc*	0.68 *ab*	2.4 *ab*	2.7 *ab*	2.8 *ab*
2	100	0.19 *c*	0.25 *b*	5.0 *ab*	3.3 *ab*	3.4 *a*
2	150	0.24 *abc*	0.47 *ab*	5.4 *ab*	2.5 *ab*	2.4 *ab*
2	200	0.26 *ab*	0.61 *ab*	4.4 *ab*	2.8 *ab*	2.3 *ab*
2	250	0.25 *abc*	0.38 *ab*	4.4 *ab*	2.9 *ab*	2.9 *ab*
3	0	0.24 *abc*	1.22 *a*	2.8 *ab*	2.3 *b*	2.2 *ab*
3	100	0.26 *abc*	1.22 *ab*	3.3 *ab*	4.6 *a*	1.9 *ab*
3	150	0.23 *abc*	0.57 *ab*	3.9 *ab*	2.5 *ab*	2.2 *ab*
3	200	0.22 *abc*	0.58 *ab*	2.5 *ab*	2.6 *ab*	2.7 *ab*
3	250	0.23 *abc*	1.04 *ab*	3.0 *ab*	2.5 *ab*	2.2 *ab*

Different letters within the columns indicate significant differences between the treatment interactions (Tukey HSD, *p* ≤ 0.05). n = 4.

**Table 5:** Effect of seed priming based on KIO_3_ and Na_2_SeO_3_ interactions on the enzymatic activity in tomato leaves.

Na_2_SeO_3_	KIO_3_	GSH	GPX	PAL	CAT	APX
(mg L^−1^)	(mg L^−1^)	(U g^−1^ TP)	(U g^−1^ TP)	(U 100 g^−1^ TP)	(U g^−1^ TP)	(U g^−1^ TP)
0	0	1.1 *cdefg*	1.0 *bcd*	13.9 *abc*	3.5 *abc*	4.6 *ab*
0	100	1.1 *bcdefg*	0.9 *cd*	12.4 *abc*	2.5 *abc*	2.1 *ab*
0	150	1.1 *bcdefg*	1.0 *cd*	11.7 *abc*	1.8 *bc*	2.4 *ab*
0	200	1.4 *a*	0.9 *cd*	13.8 *abc*	2.2 *abc*	1.5 *ab*
0	250	1.2 *abcd*	0.7 *d*	18.6 *ab*	4.1 *abc*	2.1 *ab*
0.5	0	1.0 *defg*	1.4 *abc*	19.9 *a*	2.1 *abc*	4.2 *ab*
0.5	100	1.2 *abcd*	1.1 *abcd*	17.0 *abc*	2.5 *abc*	2.1 *ab*
0.5	150	1.0 *defg*	1.8 *a*	18.7 *ab*	4.4 *abc*	4.6 *ab*
0.5	200	1.0 *cdefg*	1.7 *ab*	9.5 *c*	2.2 *abc*	3.2 *ab*
0.5	250	0.9 *g*	1.2 *abcd*	13.7 *abc*	6.3 *a*	4.2 *ab*
1	0	1.0 *cdefg*	1.0 *cd*	13.9 *abc*	3.9 *abc*	2.7 *ab*
1	100	1.2 *abcdef*	1.0 *cd*	9.5 *c*	3.5 *abc*	1.2 *ab*
1	150	1.1 *abcdefg*	1.1 *bcd*	8.7 *c*	1.8 *bc*	3.1 *ab*
1	200	1.3 *abc*	1.1 *bcd*	8.7 *c*	2.4 *abc*	3.1 *ab*
1	250	1.1 *abcdefg*	1.2 *abcd*	11.6 *abc*	4.9 *abc*	4.2 *ab*
2	0	0.9 *fg*	1.2 *abcd*	19.1 *ab*	6.1 *ab*	4.9 *a*
2	100	1.0 *defg*	0.6 *d*	10.5 *bc*	2.8 *abc*	1.6 *ab*
2	150	0.9 *efg*	0.7 *d*	10.9 *abc*	0.9 *c*	2.5 *ab*
2	200	1.1 *abcdefg*	1.3 *abcd*	12.5 *abc*	2.9 *bc*	2.8 *ab*
2	250	1.3 *ab*	1.0 *cd*	13.7 *abc*	0.7 *c*	2.8 *ab*
3	0	1.1 *bcdefg*	0.9 *cd*	12.8 *abc*	1.8 *bc*	2.2 *ab*
3	100	1.1 *abcdefg*	0.9 *cd*	11.1 *abc*	0.6 *c*	2.1 *ab*
3	150	1.2 *abcde*	0.9 *cd*	12.5 *abc*	1.7 *c*	1.2 *ab*
3	200	1.1 *abcdefg*	1.0 *cd*	12.3 *abc*	2.3 *abc*	0.8 *b*
3	250	1.1 *abcdefg*	1.1 *abcd*	12.1 *abc*	2.6 *abc*	1.4 *ab*

Different letters within the columns indicate significant differences between the treatment interactions (Tukey HSD, *p* ≤ 0.05). n = 4.

**Table 6:** Effect of seed priming based on KIO_3_ and Na_2_SeO_3_ interactions on the antioxidant capacity (µmol TE g^−1^ DW) in tomato fruits and leaves.

Na_2_SeO_3_	KIO_3_	Fruits	Fruits	Fruits	Leaves	Leaves	Leaves
(mg L^−1^)	(mg L^−1^)	ABTS-H	ABTS-L	DPPH-H	ABTS-H	ABTS-L	DPPH-H
0	0	28.7 *a*	6.6 *a*	43.7 *abc*	47.8 *ab*	20.1 *abc*	21.5 *e*
0	100	27.5 *ab*	8.4 *a*	34.1 *abc*	64.7 *ab*	14.6 *bc*	22.3 *de*
0	150	27.0 *ab*	4.0 *a*	41.0 *abc*	44.2 *ab*	17.7 *abc*	34.4 *bcde*
0	200	25.3 *ab*	3.2 *a*	42.0 *abc*	61.6 *ab*	15.8 *abc*	26.0 *cde*
0	250	27.1 *ab*	7.1 *a*	44.9 *abc*	55.3 *ab*	19.0 *abc*	36.0 *abcde*
0.5	0	20.3 *abc*	8.8 *a*	50.2 *a*	47.0 *ab*	13.1 *bc*	28.5 *cde*
0.5	100	19.3 *abc*	8.4 *a*	36.6 *abc*	56.3 *ab*	10.9 *c*	36.1 *abcde*
0.5	150	16.8 *abc*	4.7 *a*	41.3 *abc*	59.4 *ab*	10.3 *c*	40.0 *abcde*
0.5	200	26.4 *ab*	1.4 *a*	41.9 *abc*	45.7 *ab*	26.2 *a*	28.9 *cde*
0.5	250	27.4 *ab*	3.6 *a*	36.5 *abc*	42.7 *ab*	13.6 *bc*	22.3 *de*
1	0	17.3 *abc*	1.8 *a*	26.2 *abc*	52.1 *ab*	18.1 *abc*	28.0 *cde*
1	100	16.3 *abc*	2.3 *a*	35.6 *abc*	44.4 *ab*	12.7 *bc*	27.0 *cde*
1	150	27.8 *ab*	4.3 *a*	46.9 *ab*	55.3 *ab*	19.9 *abc*	48.3 *abcd*
1	200	18.1 *abc*	4.3 *a*	32.2 *abc*	44.5 *ab*	14.0 *bc*	49.7 *abc*
1	250	15.3 *bc*	2.6 *a*	32.1 *abc*	46.2 *ab*	14.0 *bc*	36.2 *abcde*
2	0	17.0 *abc*	1.7 *a*	29.4 *abc*	40.4 *b*	18.9 *abc*	36.1 *abcde*
2	100	17.2 *abc*	4.2 *a*	23.1 *b*	44.8 *ab*	19.7 *abc*	61.5 *a*
2	150	12.1 *c*	6.6 *a*	20.8 *c*	44.9 *ab*	22.5 *ab*	29.0 *cde*
2	200	10.3 *c*	3.4 *a*	28.3 *abc*	50.0 *ab*	18.9 *abc*	61.7 *a*
2	250	15.6 *abc*	2.7 *a*	26.5 *abc*	50.0 *ab*	16.4 *abc*	52.5 *abc*
3	0	18.2 *abc*	9.7 *a*	28.3 *abc*	53.0 *ab*	22.3 *ab*	56.1 *ab*
3	100	22.3 *abc*	9.6 *a*	30.7 *abc*	48.0 *ab*	19.3 *abc*	49.1 *abc*
3	150	11.8 *c*	2.3 *a*	29.4 *abc*	67.0 *a*	18.2 *abc*	42.7 *abcde*
3	200	14.9 *bc*	5.3 *a*	25.5 *abc*	54.4 *ab*	13.4 *bc*	21.6 *e*
3	250	23.2 *abc*	2.4 *a*	30.9 *abc*	57.5 *ab*	20.0 *abc*	46.9 *abcde*

-H hydrophilic, -L lipophilic. Different letters within the columns indicate significant differences between the treatment interactions (Tukey HSD, *p* ≤ 0.05). n = 4.

## Data Availability

Data shall be available through request to the corresponding author.

## Supplementary Materials

The following supporting information can be downloaded at: https://www.mdpi.com/article/10.3390/antiox12061265/s1. Table S1: List of some patented seed priming treatments commercially available; Table S2: Summary of selenoprotein functions; Figure S1: Ranges of selenium application in several crops, applications form to achieve benefits such as biofortification and stimulation; Figure S2: Biochemical and ionomic effects of selenium and nanoselenium application in plants; Figure S3: Uptake, transport and metabolism of iodine in plants; Figure S4: Iodine as a modulator of antioxidants.
