# Supplementary material for: Seed Priming Based on Iodine and Selenium Influences the Nutraceutical Compounds in Tomato (Solanum lycopersicum L.) Crop
Source: Antioxidants (Basel). 2023 Jun 13;12(6):1265. doi: 10.3390/antiox12061265 (PMC10295327; doi:10.3390/antiox12061265)

Table S1. List of some patented seed priming treatments commercially available.

Patented protocol (trading name)	Company	Description and target plants
EasyPrime<br>EasyDormex	ATLAS s.r.l.<br>(Italy)	Priming method to improve seed germination (faster, uniform, reduced abnormal seedlings). Targets: tomato, pepper, eggplant, melon, leek, Brassica.<br>Priming method to remove seed thermo- and photo-dormancy. Targets: lettuce, endive.
Advantage®<br>Xbeet®<br>Emergis®	Germains Seed Technology<br>(United Kingdom)	Priming technology for safer crop emergence, earlier plant establishment, improved root shape/size and increased stress tolerance/yield. Target: sugar beet.<br>Improved speed germination, promotes uniform emergence and stronger plant establishment. Targets: all vegetables, flowers, herbs.
Thermocure™<br>SPLITKOT®<br>ESPECIAL<br>PROMOTOR™<br>IMPROVER™	INCOTEC Europe BV<br>(The Netherlands)	Priming method to remove seed thermo-dormancy. Targets: lettuce.<br>Priming method to alleviate seed photo-dormancy in photo-sensitive varieties, increase temperature tolerance, improve germination to obtain uniform seedling establishment. Target: lettuce, endive, escarole, radicchio.<br>Priming method to improve germination efficiency/uniformity under stress conditions. Targets: onion, carrot, tomato, Brassica.<br>Primed seeds are selected based on the X-Ray image of seed interior. Targets: tomato.

Table S2. Summary of selenoprotein functions.

Selenoprotein	Abbreviations	Functions (References)
Glutathione peroxidase 1	GPX1, cytosolic glutathione peroxidase	Reduces cellular H_2O_2.
Glutathione peroxidase 2	GPX2, intestinal glutathione peroxidase	Reduces peroxide in gut.
Glutathione peroxidase 3	GPX3, Plasma glutathione peroxidase	Reduces peroxide in blood.
Glutathione peroxidase 4	GPX4, Phospholipid hydroperoxide glutathione peroxidase	Anti-oxidative lipid repair enzyme localized to cytosol, mitochondria, and nucleus, which reduces hydrogen peroxide radicals and lipid peroxides to water and lipid alcohols and prevents iron-induced cellular ferroptosis.
Glutathione peroxidase 6	GPX6	Importance unknown.
Thioredoxin reductase 1	TXNRD1, TR1	Localized to cytoplasm and nucleus and regenerates reduced thioredoxin.
Thioredoxin reductase 2	TXNRD2, TR3	Localized to mitochondria and regenerates reduced thioredoxin.
Thioredoxin-glutathione reductase	TXNRD3, TR2, TGR	Testes-specific expression, which regenerates reduced thioredoxin.
Iodothyronine deiodinase 1	DIO1, D1	Important for systemic active thyroid hormone levels.
Iodothyronine deiodinase 2	DIO2, D2	ER enzyme important for local active thyroid hormone levels.
Iodothyronine deiodinase 3	DIO3, D3	Inactivates thyroid hormone.
Methionine-R-sulfoxide reductase B1	MSRB1, SELR, SELX	Regulator of F-actin repolymerization in macrophages during innate immune response, which works in concert with MICALs to reduce oxidated methionine (R)-sulfoxide (Met-RO) back to methionine.
Selenoprotein F	SELENOF, Selenoprotein 15, SEP15	ER-resident thioredoxin-like oxidoreductase that complexes with uridine-guanosine-guanosine-thymodine (UGGT) and improves protein quality control by correcting misglycosylated/misfolded glycoproteins via the calnexin-calreticulin- endoplasmic reticulum proten 57 (ERp57) axis and pH-dependent endoplasmic reticulum proten 44 (ERp44) system.
Selenoprotein H	SELENOH, SELH, C11orf31	Nuclear localization, which is involved in redox sensing and transcription.
Selenoprotein I	SELENOI, SELI, EPT1	Involved in phospholipid biosynthesis.
Selenoprotein K	SELENOK, SELK	Transmembrane protein localized to the endoplasmic reticulum (ER) and involved in calcium flux in immune cells and ER associated degradation in cell lines.
Selenoprotein M	SELENOM, SELM, SEPM	Thioredoxin-like ER-resident protein that may be involved in the regulation of body weight and energy metabolism.
Selenoprotein N	SELENON, SELN, SEPN1	Transmembrane protein localized to ER. Mutations lead to multiminicore disease and other myopathies.
Selenoprotein O	SELENOO, SELO	Mitochondrial protein that contains a C-X-X-U (where C is cytosine, X is any nucleotide, and U is uridine) motif suggestive of the redox function.
Selenoprotein P	SELENOP, SEPP1, SeP, SELP, SEPP	Secreted into plasma for selenium transport to tissues.
Selenoprotein S	SELENOS, SELS, SEPS1, VIMP	Transmembrane protein found in ER involved in ER associated degradation.
Selenoprotein T	SELENOT, SELT	Oxidoreductase localized to the Golgi complex and ER and manifests a thioredoxin-like fold and is involved in redox regulation and cell anchorage. Complexes with UGGTs to improve PQC. Deficiency leads to early embryonic lethality.
Selenoprotein V	SELENOV, SELV	Testes-specific expression.
Selenoprotein W	SELENOW, SELW, SEPW1	Putative antioxidant role, which may be important in muscle growth.
Selenophosphate synthetase 2	SEPHS2, SPS2	Involved in synthesis of all selenoproteins including itself.

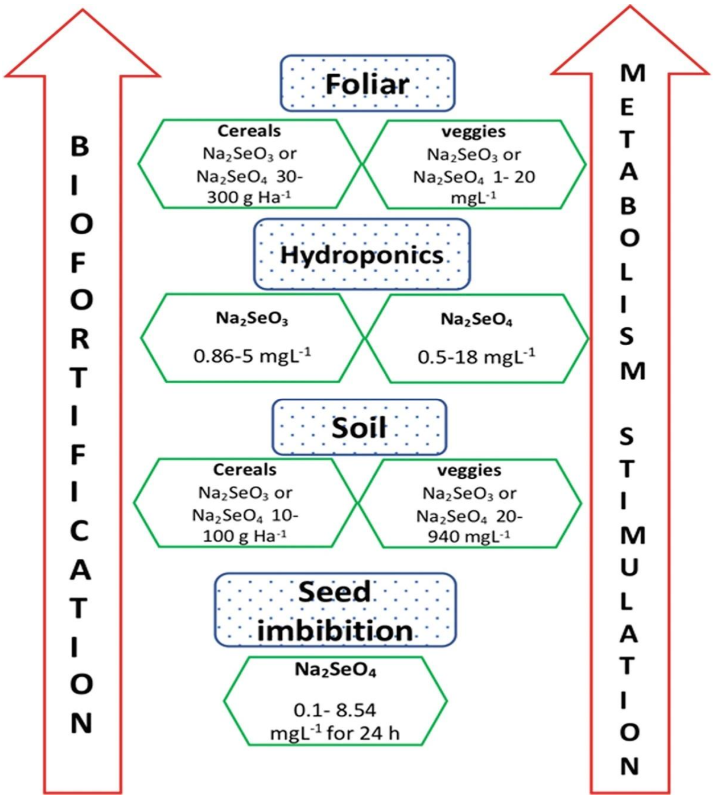

Figure S1. Ranges of selenium application in several crops, applications form to achieve benefits such as biofortification and stimulation.

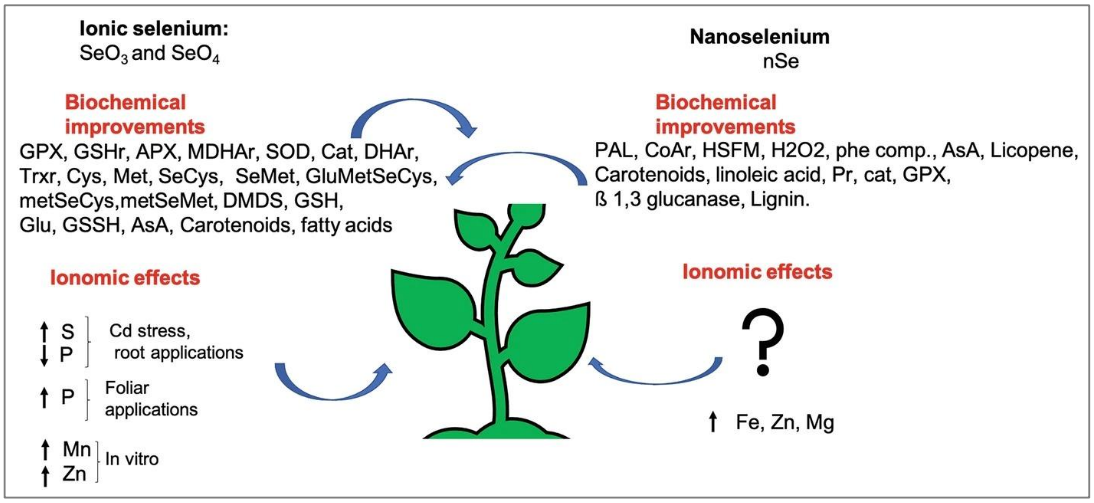

Figure S2. Biochemical and ionomic effects of selenium and nanoselenium application in plants.

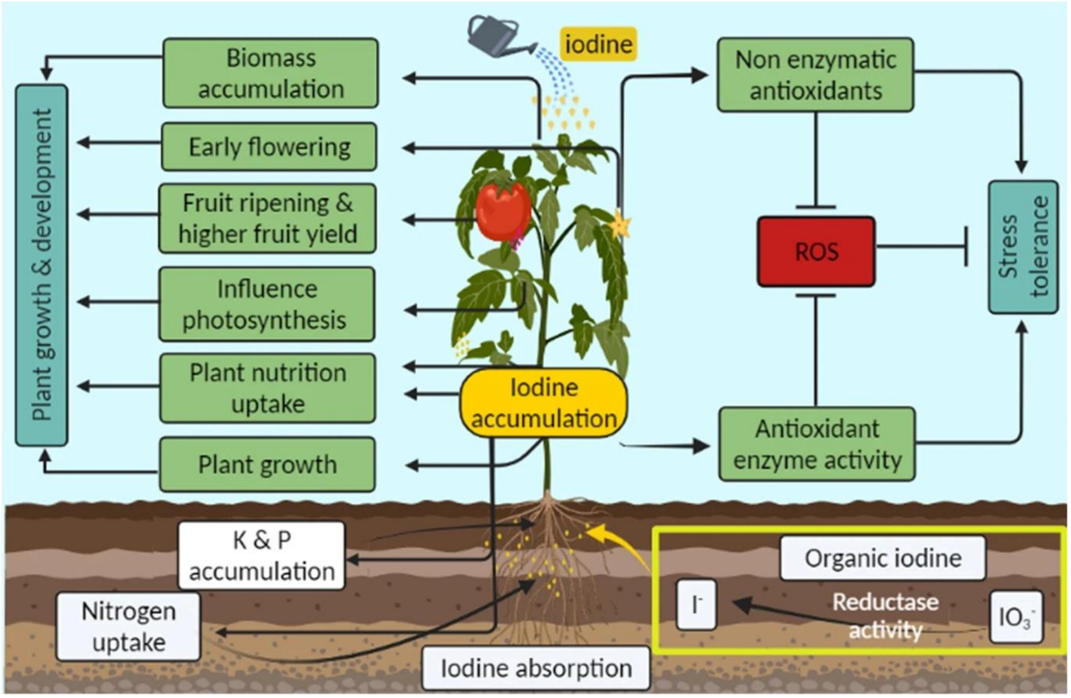

Figure S3. Uptake, transport and metabolism of iodine in plants.

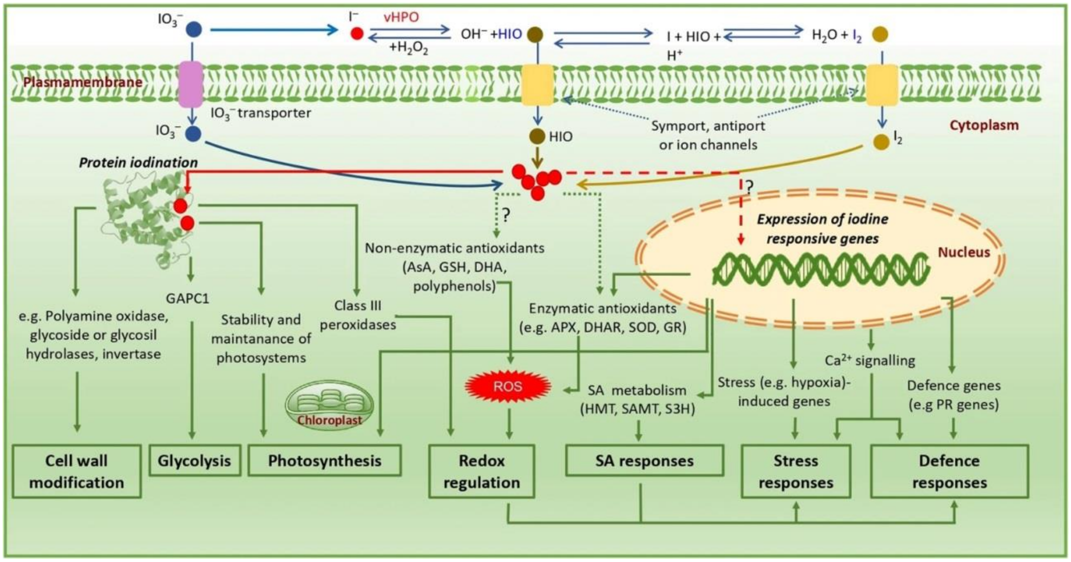

Figure S4. Iodine as a modulator of antioxidants.

Photographs of the crop throughout the time.

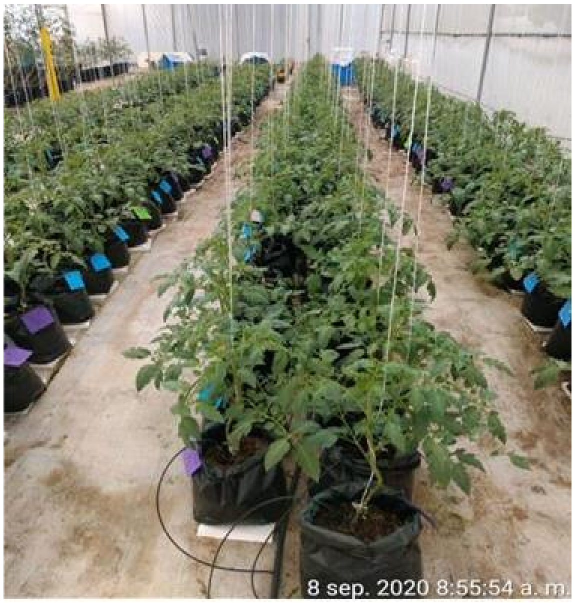

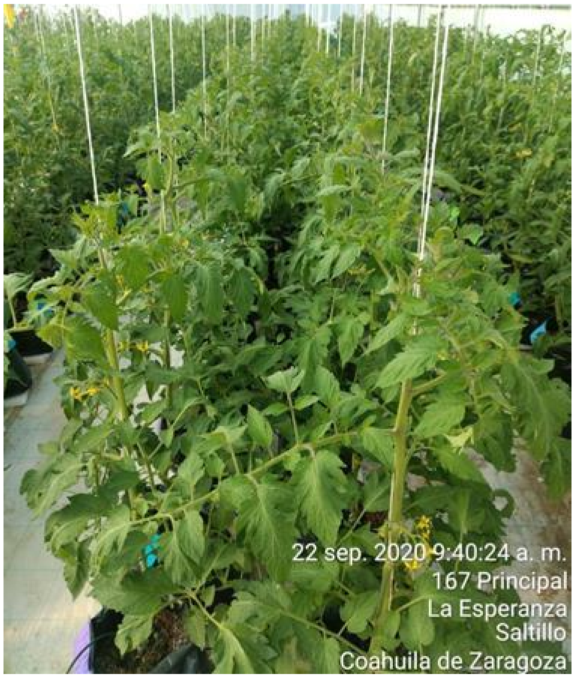

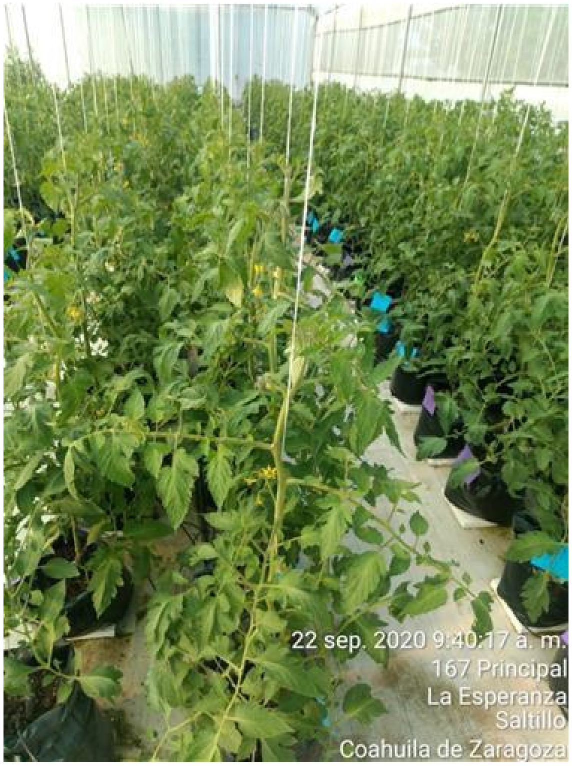

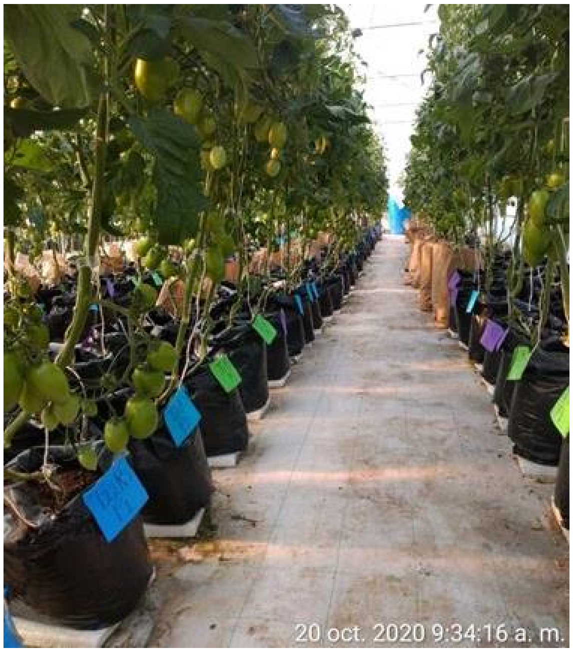
20 oct. 2020 9:34:16 a. m.

⇨

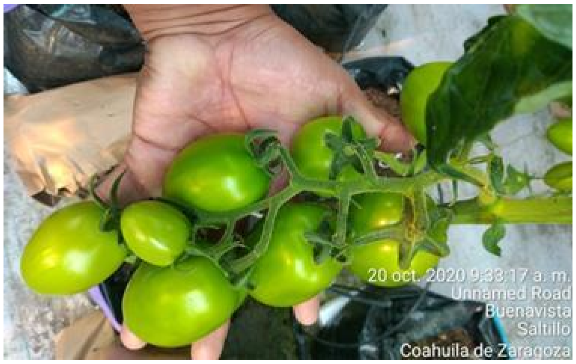
20 oct. 2020 9:33:17 a. m.
Unnamed Road
Buenavista
Saltillo
Coahuila de Zaragoza

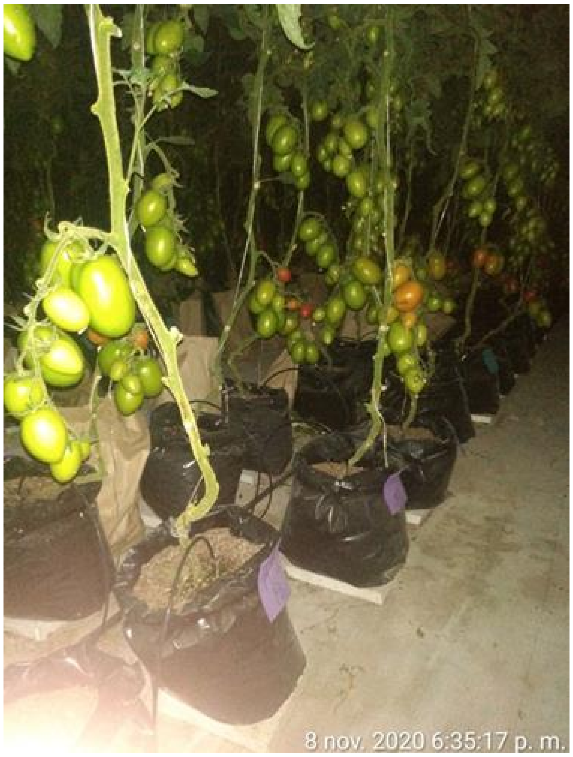
8 nov. 2020 6:35:17 p. m.

⇨

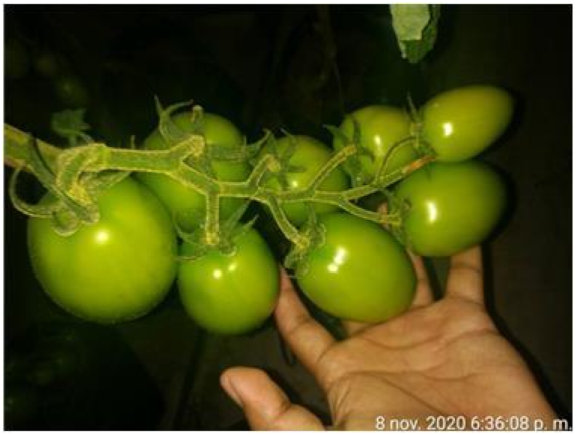
8 nov. 2020 6:36:08 p. m.

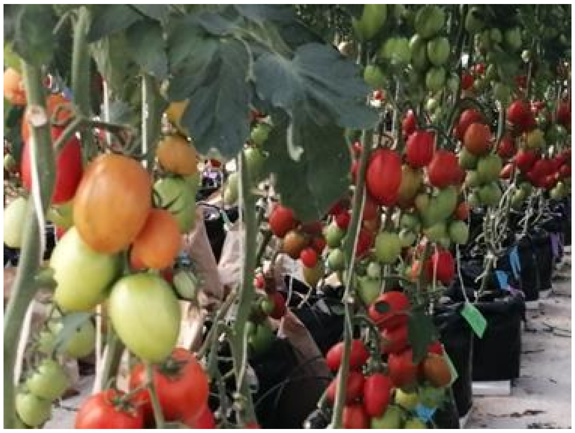

Supplement: Supplementary file 1 [file antioxidants-12-01265-s001.zip › antioxidants-2436327-supplementary.pdf]
